# The Influence of Schwann Cell Metabolism and Dysfunction on Axon Maintenance

**DOI:** 10.1002/glia.70071

**Published:** 2025-07-25

**Authors:** Rose Follis, Vishwanath V. Prabhu, Bruce D. Carter

**Affiliations:** ^1^ Vanderbilt Brain Institute, Department of Biochemistry Vanderbilt University Nashville Tennessee USA

**Keywords:** axon, myelin, Schwann cell

## Abstract

Schwann cells are the glial cells in the peripheral nervous system responsible for the production of myelin, which is essential for rapid, saltatory conduction in nerves. However, it has become increasingly recognized that Schwann cells are also key regulators of neuron viability and function, especially for sensory neurons. Neurons and Schwann cells form a tightknit, interdependent couple with complex mechanisms of communication that are only beginning to be understood. There is growing evidence that Schwann cell metabolism profoundly influences axons through the release of a variety of metabolites. These glial cells serve as energy depots for axon function, supplying lactate and/or pyruvate during repeated firing and after injury. Lipid metabolism in Schwann cells, which is critical for myelin production, also affects axon viability, such that disruptions in the production or breakdown of lipids can lead to axon dysfunction and subsequent degeneration. Here, we discuss emerging concepts on the mechanisms by which Schwann cell metabolites influence neuron activity and survival, with particular focus on how dysfunction of lipid metabolism can lead to axon degeneration and the development of peripheral neuropathy.

## Main Points

- Sensory neurons depend on Schwann cells for survival.
- Schwann cells provide energy for axons dunng rapid firing or after injury.
- Dysregulated metabolism in Schwann cells can lead to the production of neurotoxic and axon degeneration.

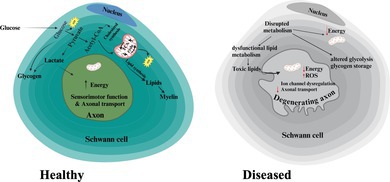

## 1 Introduction

Since their discovery in the mid‐1800s by Theodor Schwann, Schwann cells have largely been studied within the context of their, admittedly hard‐to‐ignore, roles in axon conduction and regeneration. The ensheathment of medium to large caliber peripheral axons by Schwann cells with myelin, compacted layers of specialized membrane bilayer, reduces axonal capacitance, forms the nodes of Ranvier, and guides axon channel segregation. Together, this allows for increased axonal signaling speed and efficiency through saltatory conduction and is instrumental in the organization and honing of the peripheral nervous system (PNS) (Jessen et al. 2008; Mirsky et al. 2002). Consequently, the importance of Schwann cells to PNS pathobiology cannot be overstated, with numerous neuropathies either caused or perpetuated by dysfunctional myelin formation and/or maintenance (Chrast et al. 2011; Duncan and Radcliff 2016; Li 2015). Equally, the ability of Schwann cells to reprogram into precursor‐like cells after nerve injury and to promote regeneration far beyond what is achievable by their central nervous system (CNS) counterparts, oligodendrocytes, has been instrumental in advancing CNS regenerative therapies (Jessen and Mirsky 2016).

Emerging evidence indicates that Schwann cell metabolic dysfunction may significantly contribute to many peripheral nerve pathologies, potentially due to the accumulation of neurotoxic lipid species or glycolytic metabolites. This review summarizes recent findings linking Schwann cell metabolic dysfunction to a broad range of inherited and acquired neuropathies.

## 2 The Impact of Neuropathy

Neuropathy, sometimes also referred to as peripheral neuropathy, is defined as any disorder characterized by peripheral nerve abnormalities, whether these abnormalities are inherited (genetic), acquired (metabolic/toxic insult), or idiopathic (of unknown origin). Common abnormalities include structural alterations to peripheral neurons (axon degeneration and metabolic/conductive disruption), Schwann cells (dys‐/demyelination), or both (Azhary et al. 2010; Hughes 2002). These abnormalities typically result in electrophysiological deficits that impair nerve conduction. Depending on the severity and type of neuropathy, these disorders can result in neuropathic pain, loss of sensation, muscle weakness, autonomic dysregulation, and skeletal abnormalities. In the United States, it is estimated that 14.8% of persons over 40 have neuropathy, with that number expected to increase due to the rising prevalence of Type 2 diabetes mellitus (Gregg et al. 2004). Diabetic neuropathy is not only the most common form of acquired sensory neuropathy, but a leading risk factor for severe diabetic complications (Hicks and Selvin 2019). Therefore, the development of new treatments for neuropathies is of both medical and economic importance, especially given the rising global median age. Unfortunately, our understanding of the complex pathological mechanisms underlying neuropathy is far from complete. Additionally, while clinical presentation and diagnosis of many neuropathies occur in adulthood, the underlying etiology is often present years before diagnosis, in many cases even during PNS development when the survival and establishment of neurons and Schwann cells are heavily interdependent, making a more complete understanding of the complex nature of peripheral nerve formation and metabolism a key priority in advancing treatment discovery (Azhary et al. 2010).

## 3 Schwann Cell Development

Almost all peripheral glial cells and neurons (autonomic and somatosensory) originate from neural crest cell progenitors. Dorsal root ganglia (DRG) neurons begin development as clear forerunners; Schwann cell precursors do not infiltrate the peripheral nerve until around E12–13 (Jessen and Mirsky 2019). Glial cell populations remaining within the DRG after migration, and associated with somata rather than axons, become satellite glia (Hanani and Spray 2020; Le Douarin and Dupin 1993). Once present in the nerve, Schwann cell precursors are reliant on axonal signals for migration guidance, continued proliferation, and survival signaling as they migrate in association with pioneer target‐seeking axonal projections from DRG neurons or from spinal motor neurons (Mirsky et al. 2008).

Around E15–16, Schwann cell precursors (SCPs) in the nerve, responding to persistent axonal signaling, including but not exclusively in the form of neuregulin I (NRG1) signaling through the ErbB (erb‐b receptor tyrosine kinase) 2/3 receptor complex, begin transcriptional remodeling that results in a transition to autocrine competent, basal lamina secreting immature Schwann cells (Taveggia and Feltri 2022). Beginning perinatally, these immature Schwann cells begin to sort and subdivide axons into organized bundles, or fascicles, segregating large and small caliber axons, in a multi‐stage process called radial sorting. Immature Schwann cells that are associated in a 1:1 relationship with medium and large caliber axons during the later stages of radial sorting then begin a transition to pro‐myelinating Schwann cells (Feltri et al. 2016). Pro‐myelinating Schwann cells are driven to become mature, myelinating cells, in part, by axonal NRG1, particularly the membrane‐bound type 3 isoform. This differentiation process involves the activation of a number of transcription factors such as Sox10 (Britsch et al. 2001; Kuhlbrodt et al. 1998), Oct6 (Bermingham et al. 1996; Jaegle et al. 1996), NFAT (Kao et al. 2009; Kipanyula et al. 2013), NF‐kB (Nickols et al. 2003; Yoon et al. 2008) and Krox20 (Nagarajan et al. 2001; Topilko et al. 1994). After birth, and concurrent with the last stages of radial sorting, myelination begins and continues until approximately the third week of life (Mirsky et al. 2008). In contrast, some immature Schwann cells differentiate into non‐myelinating Schwann cells, which ensheath groups of small caliber axons in what are referred to as Remak bundles. The mechanisms promoting Remak Schwann cell formation and differentiation are not well understood (Harty and Monk 2017). For a more comprehensive review of Schwann cell development and myelin formation please see (Jessen and Mirsky 2019; Monk et al. 2015; Muppirala et al. 2021).

## 4 Lipid Metabolism in Developing Schwann Cells

As part of the differentiation process, myelinating Schwann cells undergo an immense metabolic reorganization and increase in lipid biosynthesis. It is estimated that the plasma membrane of Schwann cells expands 6500 fold during myelination (Webster 1971). Additionally, myelin has a much larger ratio of lipids to proteins than average cell membranes, with lipids constituting 70%–80% of compact myelin by weight relative to 50% in other cells, and a very specialized lipid composition (Poitelon et al. 2020). While there are no myelin‐specific lipids, several classes of lipids are highly enriched in peripheral nerves including cholesterol (~26% of myelin), glycolipids (~31%, most notably galactosylceramide), and sphingomyelin phospholipids (~10%–35%) (Chrast et al. 2011; Garbay et al. 2000).

Furthermore, while lipid uptake from the bloodstream, especially cholesterol, often reduces metabolic demands on many cells, the lipids of myelin membranes are theorized to be primarily synthesized *de novo* in Schwann cells (Fu et al. 1998; Jurevics et al. 1998). Multiple studies have shown that reducing or altering cholesterol or fatty acid synthesis in developing Schwann cells results in peripheral myelin deficits, including early postnatal lipid depletion and hypomyelination. These conditions typically can only be partially rectified by increased external uptake later in development (Montani et al. 2018; Saher et al. 2005; Verheijen et al. 2009). Additionally, the low‐density lipoprotein receptor LDLR, which is most associated with exogenous cholesterol uptake, is dispensable for both myelin generation and repair in the peripheral nerve (Goodrum et al. 1994, 2000), suggesting that, as with oligodendrocytes in the CNS, Schwann cells are not primarily reliant on hemodynamically circulated lipids.

This endogenous lipid production may be necessary due to the presence of the blood–nerve barrier (BNB), a system analogous to the more well‐studied blood–brain barrier (BBB) of the CNS. The BNB consists of the perineurium, a multilayered structure surrounding bundles of axons, or fascicles, and the vessels of the endoneurium which are sealed by tight junction‐expressing endothelial cells and pericytes at endoneurial capillaries (Reinhold and Rittner 2020). The BNB is impermeable to many lipids and other large molecules; therefore, it likely limits the rate and efficiency of exogenous lipid uptake for Schwann cells from peripheral circulation. However, lipid permeability during development and myelination is not well characterized, although it has been noted that endoneurial tight junctions are less robust at birth (Reinhold and Rittner 2020). Additionally, the DRGs as well as their dorsal roots are excluded from the BNB, making it difficult to determine the true extent of DRG neuron and Schwann cell isolation from broad hemodynamic metabolism (Cesmebasi 2015).

The production of lipids by Schwann cells for myelin formation is not only critical from a structural perspective, but also for signaling. Many lipids are part of signal transduction pathways that regulate myelination. Lysophosphatidylcholine (LPC) has long been used to induce focal demyelination in peripheral nerves (Hall and Gregson 1971) and is thought to be one of the signals promoting Schwann cell demyelination and dedifferentiation following nerve injury (Nagai et al. 2010). Recently, Hu et al. demonstrated an elegant feedback system by which lipids synthesized during myelination can serve to promote or inhibit myelin formation through p21(Rac1)‐activated kinase, PAK2 (Hu et al. 2024). Gene deletion of PAK2 revealed that it is essential for axon sorting by Schwann cells during myelin formation. Notably, several myelin‐enriched lipids, such as cholesterol ester, phosphatidylinositol, or GM3 ganglioside, activated PAK2, while sphingosine inhibited it. Thus, the generation of components of the myelin membrane can serve as a feedback mechanism to regulate its formation.

Even though endogenous lipid biosynthesis is a key component of myelinating Schwann cell development, our understanding of the modulators of lipid metabolism in the PNS is limited. There is some evidence that the timing of lipid biosynthesis during myelination, as with myelin proteins, is regulated primarily by Schwann cell‐axonal signaling in the PNS. NRG1, produced by axons, has been linked to the transcription of HMG‐CoA‐reductase (3‐hydroxy‐3‐methylglutaryl‐coenzyme‐A‐reductase), the rate‐limiting enzyme of cholesterol biosynthesis, and the regulation of Maf and mTOR, two broadly focused metabolism‐governing kinases (Kim et al. 2018; Pertusa et al. 2007; Poitelon et al. 2020). Deletion of mTOR in murine Schwann cells resulted in thinner myelin and shorter internodes, consistent with disrupted lipid catabolism, although this was not directly investigated (Sherman et al. 2012). mTOR forms two large protein complexes, mTORC1 and mTORC2, that regulate many aspects of cellular metabolism and growth, including lipid biosynthesis. Disruption of mTORC1, but not mTORC2, in Schwann cells of mice resulted in reduced expression of the transcription factor Sterol‐responsive element binding protein 1c (SREBP1c), which caused dysregulation of many lipid genes. The mice with this dyslipidemia exhibited hypomyelination and decreased nerve conduction, highlighting the importance of this signaling pathway (Norrmén et al. 2014).

Another axonal signal, BDNF, acting through the p75 neurotrophin receptor (p75NTR) on Schwann cells, promotes cholesterol biosynthesis by activating another Sterol‐responsive element binding protein, SREBP2 (SREBP2), which controls sterol and lipid metabolism (Follis et al. 2021). Additionally, Krox20, a transcription factor instrumental in the upregulation of many myelin genes, also acts synergistically with SREBP1 and SREBP2 to promote the induction of a variety of lipid biosynthetic genes during Schwann cell differentiation into a myelinating phenotype (LeBlanc et al. 2005).

## 5 Neuronal Dependence on Schwann Cells

### 5.1 Developmental Dependence on Schwann Cells

**FIGURE 1:**
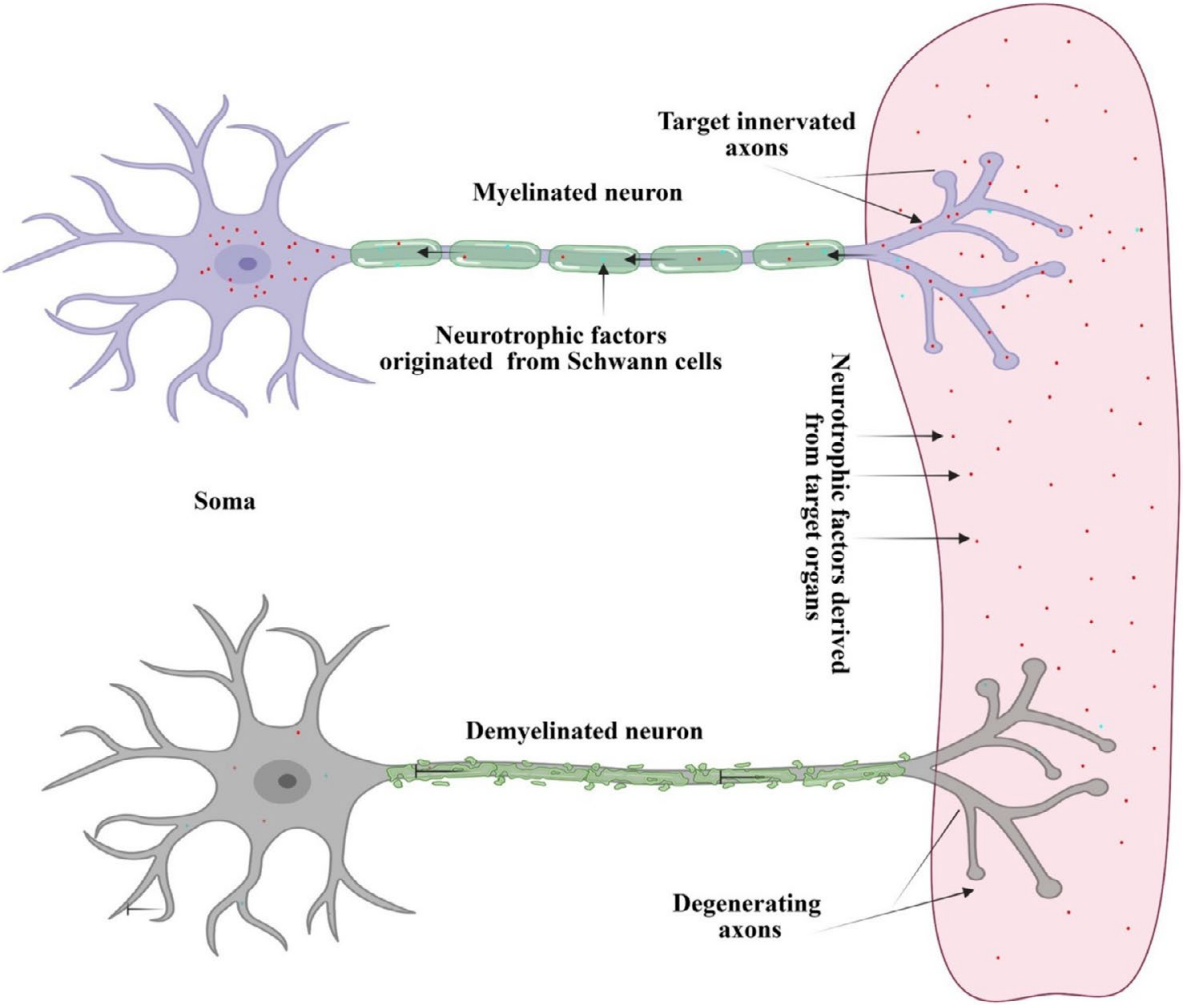
Schwann cells may provide trophic support for neurons prior to final target innervation. Neurotrophic factors derived from Schwann cells and target organs are shuttled to the neuron soma via retrograde axonal transport to promote survival. Loss of Schwann cells or disruption of their axon association results in reduced trophic signaling, leading to degeneration.

Evidence suggests that during early development, sensory and spinal motor neurons depend on Schwann cells for their survival prior to final target innervation (Figure 1) (Davies 1998). For example, studies investigating the role of NRG1 signaling in Schwann cell development revealed a dependence of DRG neurons on these glial cells. In mice with inactivating mutations in components of the neuregulin signaling pathway, SCPs are generated but soon die off due to the lack of this critical trophic factor. It was noted that the DRG neurons form normally in these mice and project out to their targets, but eventually undergo apoptosis due to the glial cell loss (Garratt et al. 2000; Riethmacher et al. 1997; Woldeyesus et al. 1999; Wolpowitz et al. 2000). Likewise, deficits in both early and late stages of radial sorting cause neuropathy in humans as well as animal models. For example, disruption of the *LAMA2* gene encoding Laminin alpha‐2 disturbs basal lamina formation in immature Schwann cells and results in the dysmyelinating neuropathy and muscular dystrophy, Mersin‐deficient Congenital Muscular Dystrophy, characterized by a preferential loss of large diameter sensory and motor fibers (Di Muzio et al. 2003).

The mechanisms by which Schwann cells promote neuron viability during development are not well understood. However, these glial cells produce a variety of neurotrophic factors, such as BDNF, NT3, GDNF, and CNTF, which may provide key survival signals for neurons en route to their final targets (Davies 1998) (Figure 1). Schwann cells are also essential for the formation of polarized domains in axons; in particular, the nodes of Ranvier and the associated paranodal and juxtaparanodal regions. The nodes are where voltage‐gated sodium channels cluster, while voltage‐gated potassium channels are localized to the juxtaparanode. Schwann cells regulate the formation of these regions through secretion of matrix factors as well as through direct contact between transmembrane proteins on the Schwann cell and the axon (Eshed‐Eisenbach and Peles 2013; Elson and Jenkins 2017). These signals result in the reorganization of the axonal cytoarchitecture, including the localization of mitochondria to the nodes, which help power the generation of action potentials. Disruption of nodal formation changes the axonal structural organization and can affect mitochondrial localization and transport, which have been implicated in subsequent axon degeneration (Moss et al. 2021).

### 5.2 Dependence on Schwann Cells for Energy in Adulthood

Primary dys‐/demyelination of Schwann cells followed by secondary axonal degeneration is a common feature of many inherited and acquired neuropathies, suggesting that Schwann cell support and/or myelin integrity is necessary for peripheral neuron function throughout adulthood (Azhary et al. 2010). Intuitively, perhaps it is not surprising that these glial cells could serve as a fuel reservoir for their associated axons. Many peripheral neurons have very long axons, up to 1 m in humans, with high energetic demands in order to propagate action potentials. Schwann cells ensheathing the axons could serve as a convenient local energy storage depot; however, demonstrating this concept has proven challenging. Brown et al. (2012) reported that Schwann cells store glycogen and if its breakdown was inhibited during aglycemia, then conduction failed in large myelinated fibers (Brown et al. 2012). Moreover, they found lactate release into the extracellular space in the presence of glucose, but this decreased during aglycemia, leading the authors to suggest that Schwann cells release lactate to support myelinated axons. This group recently used an ex vivo nerve preparation to demonstrate that repeated stimulation of the sciatic nerve depleted Schwann cell glycogen stores, ultimately resulting in compound action potential failure. However, repeated firing of the nerves could be maintained if a high level of glucose was present in the bath, providing evidence for axon dependence on Schwann cell energy stores for repeated action potential propagation (Rich et al. 2022). Further support for this idea was provided by the Tricaud group through deletion of pyruvate kinase 2 (PKM2) (Deck et al. 2022). PKM2 is a master regulator of glycolysis and can promote the production of lactate from pyruvate. When PKM2 was selectively deleted in myelinating Schwann cells, there was a drop in Schwann cell lactate, but axonal lactate levels were only reduced in the mutant mice after repeated electrical stimulation.

In addition to the acute need of axons for Schwann cell‐derived lactate during repeated firing, there also appears to be a chronic dependence on energy provided by the glia. The PKM2 null mice exhibited a progressive reduction in motor function, indicated by rotorod and grip strength tests, although the number of motor neurons did not decrease and Schwann cell viability did not appear to be affected, and myelin remained fully intact. Axonal conduction properties were also not altered; however, there was a reduction in mitochondrial movement in the axons and denervation of neuromuscular junctions, suggesting distal neuropathy. Thus, long‐term deprivation of Schwann cell‐derived lactate results in distal neuropathy, possibly through limiting mitochondrial transport.

In the CNS, it is well established that oligodendrocytes, which are the CNS myelinating cells, similarly provide essential energetic support to axons, primarily through the transfer of lactate (Lee et al. 2012; Fünfschilling et al. 2012). This transfer occurs via monocarboxylate transporters (MCT) on the oligodendrocytes and axons. The CNS axons appear to be more dependent on this glial energy than those in the periphery, as disruption of lactate shuttling by deletion or silencing of MCT1, which is highly expressed by oligodendrocytes, resulted in axon degeneration and neuron loss in the CNS (Lee et al. 2012). MCT1 is also expressed by Schwann cells but, in contrast to results in the CNS, deficiency (Morrison et al. 2015) or even complete deletion of the transporter selectively in Schwann cells (Jha et al. 2020; Bouçanova et al. 2021) did not result in neuronal death or reduced nerve conduction. It is possible that redundant mechanisms exist for shuttling metabolic precursors, such as other MCTs; however, there was denervation of the motor endplate, reminiscent of the PKM2 knockout mice, corroborating the need for lactate from the Schwann cells for long‐term maintenance of axon health (Bouçanova et al. 2021).

Curiously, excess lactate production by Schwann cells appears to be detrimental to axons. Jia et al. recently deleted the small GTP binding protein Rheb, selectively in Schwann cells, and observed minimal effects on myelin but progressive axon degeneration (Jia et al. 2021). They found that the loss of Rheb led to a reduction in pyruvate dehydrogenase activity, resulting in increased production of lactate by the Schwann cells. Limiting axonal access to the lactate by reducing glycolysis with 2‐deoxyglucose or adding an MCT1 inhibitor rescued the axons. The authors suggested that persistent metabolism of lactate by neurons resulted in oxidative stress, reducing axon stability. A similar increase in lactate release by Schwann cells leading to axon degeneration was reported following glial deletion of LKB1, an upstream regulator of AMP kinase, which is a critical regulator of energy homeostasis (Beirowski et al. 2014). Interestingly, reminiscent of diabetic neuropathy, only sensory axons were affected; the motor neurons were preserved, although the mechanisms underlying this difference were not addressed. In contrast to the Rheb knockout mice, reducing glycolysis with 2‐deoxyglucose in the LKB1 null animals exacerbated the axon loss. This result led the authors to conclude that the release of lactate reflected an effort by the LKB1 deficient Schwann cells to protect the axons. However, it is important to note that deleting LKB1 in Schwann cells also affected lipid metabolism, which could result in the production of neurotoxic lipids that influence the response to inhibiting glycolysis. The underlying mechanisms accounting for the degeneration of sensory axons following deletion of LKB1 remain to be determined. Nevertheless, these studies both highlight the tight coupling between glial metabolism and axon integrity and emphasize our poor understanding of the signaling mechanisms involved.

In addition to axonal reliance on Schwann cells for energy during normal function, there is accumulating data that the transfer of lactate or its precursor, pyruvate, is essential for axon viability and regeneration following nerve injury. It has been recognized for some time that axotomy results in energy depletion in the distal axons and that supplementing the damaged axon with glycolytic products such as pyruvate can prevent the breakdown of the distal portion (a process referred to as Wallerian degeneration) (Wang et al. 2005). More recently, Babetto et al. demonstrated a shift in Schwann cell metabolism to glycolysis following nerve crush, driven by an mTOR‐HIF1α‐cMyc pathway (Babetto et al. 2020). The enhanced glycolysis was accompanied by an increase in lactate release and upregulation of MCT1 and 4 transporter expression. Moreover, knocking down components of the glycolytic path or pharmacologically blocking MCTs resulted in more rapid axon degeneration after the injury, suggesting that the Schwann cells were transferring protective metabolites to the axons via the transporter. Indeed, the addition of a cell‐permeable derivative of pyruvate to severed axons in vitro was sufficient to prevent the normal degradation process from occurring. Similarly, Morrison et al. found delayed nerve regeneration and reduced muscle innervation after crush injury in MCT1 heterozygous mice (Morrison et al. 2015). One mechanism by which damaged axons may activate energy transfer from Schwann cells is through the ability of Schwann cells to phagocytose the debris after injury. Recent findings demonstrated that Schwann cells deficient in the engulfment receptor TREM2 had reduced mTOR signaling due to activation of AMP kinase, which resulted in impaired regeneration after nerve crush in a mouse model of acute motor axonal neuropathy (Zhang et al. 2024). Thus, Schwann cell‐derived lactate or pyruvate not only protects damaged axons but also promotes the growth of regenerating fibers.

Altered levels of lactate have also been observed in some neuropathies; most notably, increased lactate has been observed in models of diabetic neuropathy. Rats treated with streptozotocin to induce diabetes exhibited elevated lactate in their sciatic nerves (Stevens et al. 2000; Obrosova et al. 1999). While the accumulation of lactate could be an unrelated consequence of the neuropathy, when mice with reduced expression of MCT1 were treated with streptozotocin, the neuropathy that developed was enhanced relative to wild‐type mice (Jha et al. 2020). This result suggests that the increased lactate levels are beneficial and may reflect the Schwann cells efforts to support their associated axons. Certainly, further investigation into the role of Schwann cell metabolism in diabetic neuropathy is warranted.

## 6 Schwann Cell Lipid Dysregulation and Neuropathy

Not only is Schwann cell production of lactate key to axon health, the metabolism of lipids also significantly impacts axons. Disruption of Schwann cell lipid biosynthesis and/or breakdown can drastically affect neurons, not just due to loss of trophic support by Schwann cells or myelin impairment, but also due to the accumulation of toxic lipid species within the nerve, leading to axon degeneration/dysfunction; for example, when mitochondrial dysfunction was induced selectively in Schwann cells by deletion of the mitochondrial transcription factor, Tfam (Viader et al. 2011, 2013). Loss of Tfam caused a shift from fatty acid production to oxidative breakdown of lipids in the Schwann cells. However, this catabolic process could not be completed due to depletion of NAD+ (a result of the mitochondrial dysfunction), thus there was a buildup of fatty acid intermediates, acyl‐carnitines. The Schwann cells released the acyl‐carnitines, which were readily transferred to axons, given their intimate association. These lipids altered ion homeostasis, causing excess levels of Ca^2+^ in the axons, which triggered severe axon degeneration. Notably, the neuropathy in the mice lacking Schwann cell Tfam was very similar to what is observed in diabetic neuropathy. Moreover, accumulation of circulating long‐chain free fatty acids often occurs in the progression toward type 2 diabetes and such lipid buildup induces mitochondrial dysfunction and oxidative stress (James et al. 2021; Perez‐Matos et al. 2017).

A similar degeneration of neurons due to excess long chain fatty acids is observed upon loss of acyl‐CoA oxidase 1 (ACOX1). This enzyme catalyzes the first and rate‐limiting step for β‐oxidation of very long‐chain fatty acids (VLCFAs) and is enriched in glia, including Schwann cells, with minimal expression in neurons (Chung et al. 2020). Deficiency of ACOX1 in humans results in accumulation of VLCFAs and severe neurological defects, with most patients not surviving past early childhood (El Hajj et al. 2012; Ferdinandusse et al. 2007). The mechanisms by which excess VLCFAs cause neurodegeneration are not well understood but do lead to glial cell loss, which likely affects neuron viability. Interestingly, a mutation in ACOX1 (N237S), also found in some patients, acts in a dominant gain‐of‐function manner, producing excess hydrogen peroxide as a by‐product of the enzymatic activity. The H_2_O_2_ generates reactive oxygen species (ROS), leading to neurodegeneration (Chung et al. 2020). Thus, loss‐ or gain‐of‐function of this key fatty acid catabolic enzyme can lead to neurodegenerative conditions.

Disrupting sterol production in Schwann cells can also cause neurodegeneration. As discussed above, p75NTR regulates cholesterol biosynthesis and loss of the receptor in Schwann cells resulted in the accumulation of the cholesterol precursor, 7‐dehydrocholesterol (7‐DHC), a highly reactive, neurotoxic oxysterol. The excess production of 7‐DHC did not affect Schwann cell viability, but led to the loss of approximately 30% of the sensory neurons during development, resulting in sensory deficiencies in these mice (Follis et al. 2021). Similarly, deficiency in 7‐dehydrocholesterol reductase, which converts 7‐DHC to cholesterol, is associated with the neurodegenerative syndrome, Smith–Lemli–Opitz (Porter and Herman 2011), although this condition has primarily been studied for its effects in the CNS.

It has been well established that the accumulation of neurotoxic lipids in the brain contributes to many neurodegenerative pathologies, such as Alzheimer's Disease, Parkinson's Disease, Multiple Sclerosis, and several inherited leukodystrophies (Corraliza‐Gomez et al. 2019). Additionally, the many neurodegenerative diseases tied to dysfunctional regulation or disruption of lipid metabolism, especially those that involve lipid accumulation, heavily favor pathways that are highly enriched in myelinating cells (Chrast et al. 2011; Garbay et al. 2000). In many of these disorders, the accumulation of these neurotoxic lipid species has been noted not just within the brain, but also within peripheral nerves and tied to neuropathic symptomatology (Dali et al. 2015; Dodge 2017). This has led to the exploration of the contribution of Schwann cell metabolic dysfunction to toxic gain‐of‐function pathology in a wide range of inherited and acquired neuropathies.

**FIGURE 2:**
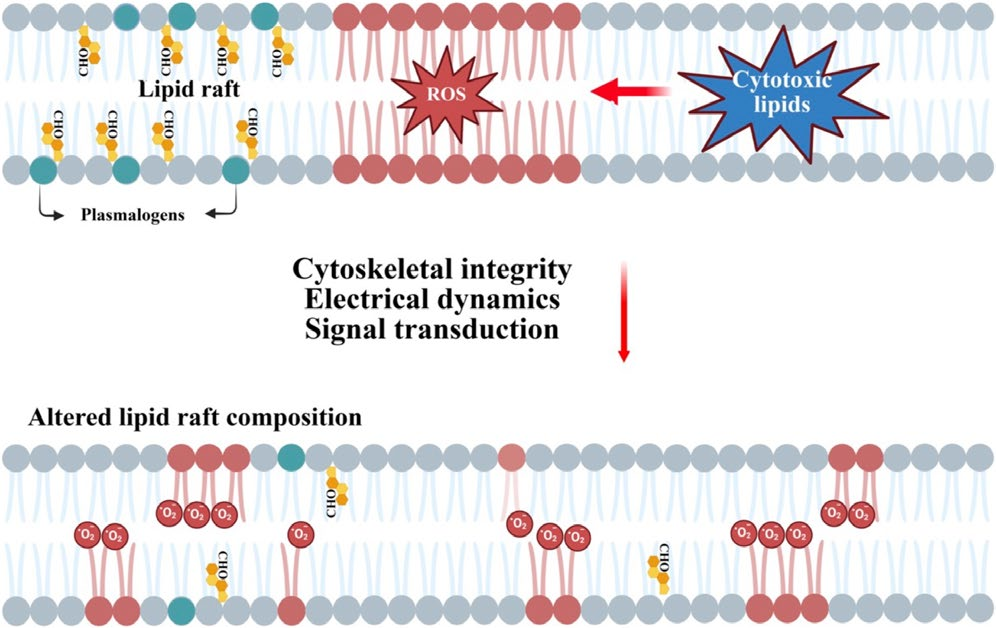
Destabilization of membrane functions by neurotoxic lipids. Disrupting the delicate balance of lipid composition through deficiency in key lipid species (e.g., plasmalogens (shown in teal)), oxidative modification of lipids, or incorporation of cytotoxic lipids can alter membrane fluidity and permeability, thereby disrupting lipid rafts, which are major centers for vital processes such as signal transduction, nutrient transport, and cell‐to‐cell communication, contributing to neurodegenerative conditions. (CHO, cholesterol).

**FIGURE 3:**
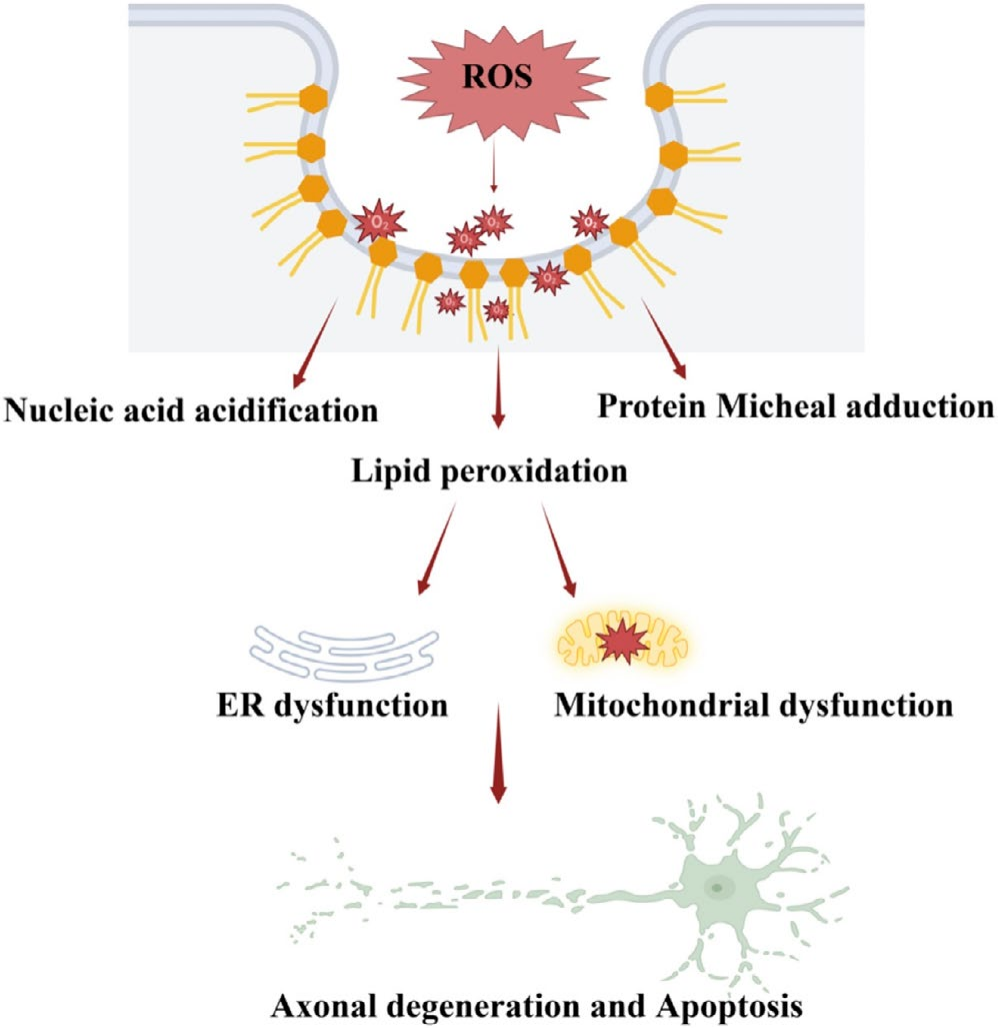
Initiation of oxidative stress by cytotoxic lipids contributes to ROS generation and lipid peroxidation. Oxidative stress begins when harmful cytotoxic lipids trigger reactions that produce reactive oxygen species (ROS). These reactive molecules can damage cellular structures like proteins, DNA, and lipids. This leads to lipid peroxidation, where the lipids in cell membranes are oxidized, compromising their integrity and functionality. This cycle exacerbates cellular damage, potentially resulting in axon degeneration and cell death.

**FIGURE 4:**
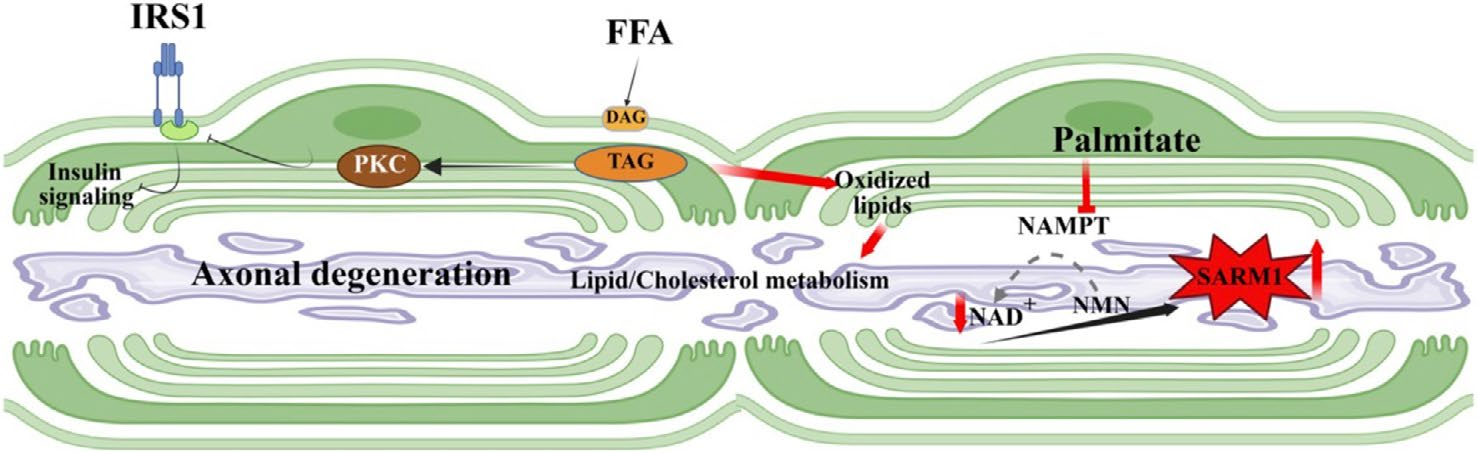
The buildup of harmful lipids like di‐ and triacylglycerides (DAGs and TAGs) or fatty acids, like palmitate, causes a disturbance in energy metabolism, ultimately leading to the deterioration of axons. DAGs and TAGs can activate protein kinase C (PKC), which phosphorylates and inhibits components of the insulin receptor signaling pathway, causing reduced glucose metabolism and, ultimately, axon degeneration. Certain lipids, such as palmitate, can suppress the production of NAD+, resulting in SARM1 activation, which results in axon degeneration.

**FIGURE 5:**
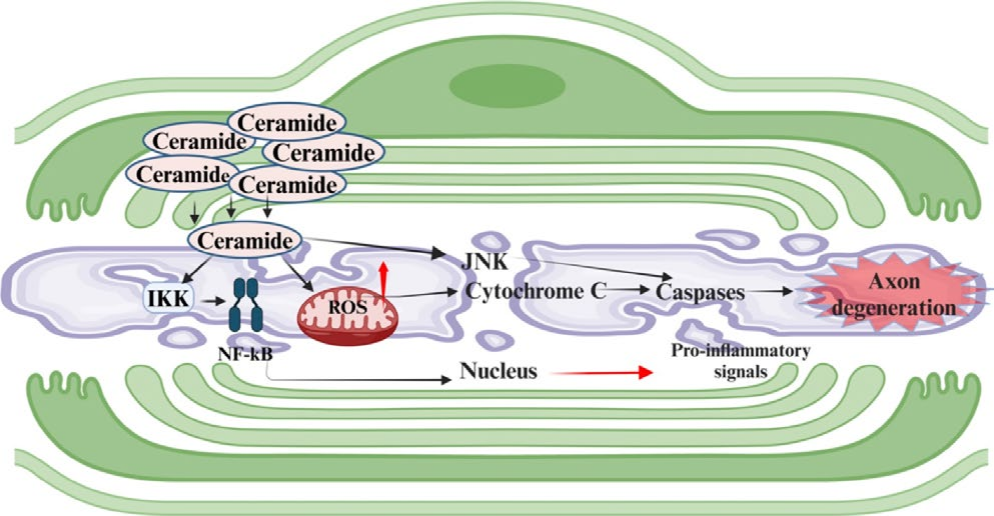
Certain lipids, particularly ceramides, can act as signaling molecules that stimulate pathways promoting neurodegeneration. As ceramides accumulate, they can stimulate inflammatory pathways, such as NF‐κB, the stress kinase JNK, which can activate caspases, and damage mitochondria, leading to the production of reactive oxygen species (ROS) and the release of cytochrome C, further activating caspases. These pathways all ultimately promote axon degeneration.

**FIGURE 6:**
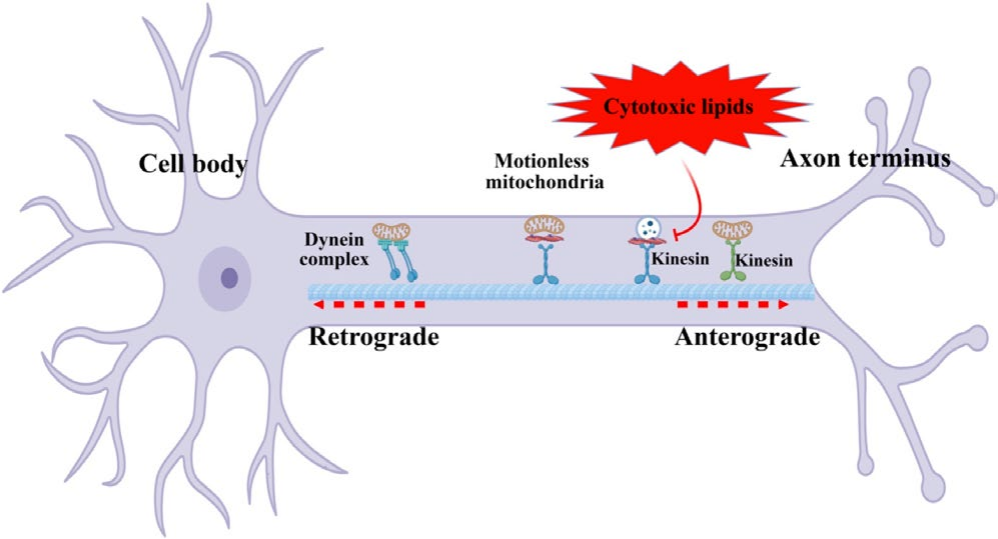
Cytotoxic lipids can disrupt the delicate balance of axonal transport. These damaging molecules can disrupt transport either directly, by altering the function of the motor proteins dynein and kinesin, or indirectly, by impairing mitochondrial function and energy generation. Preventing axonal transport leads to a breakdown in cellular communication and function, resulting in axon degeneration and cell death.

**FIGURE 7:**
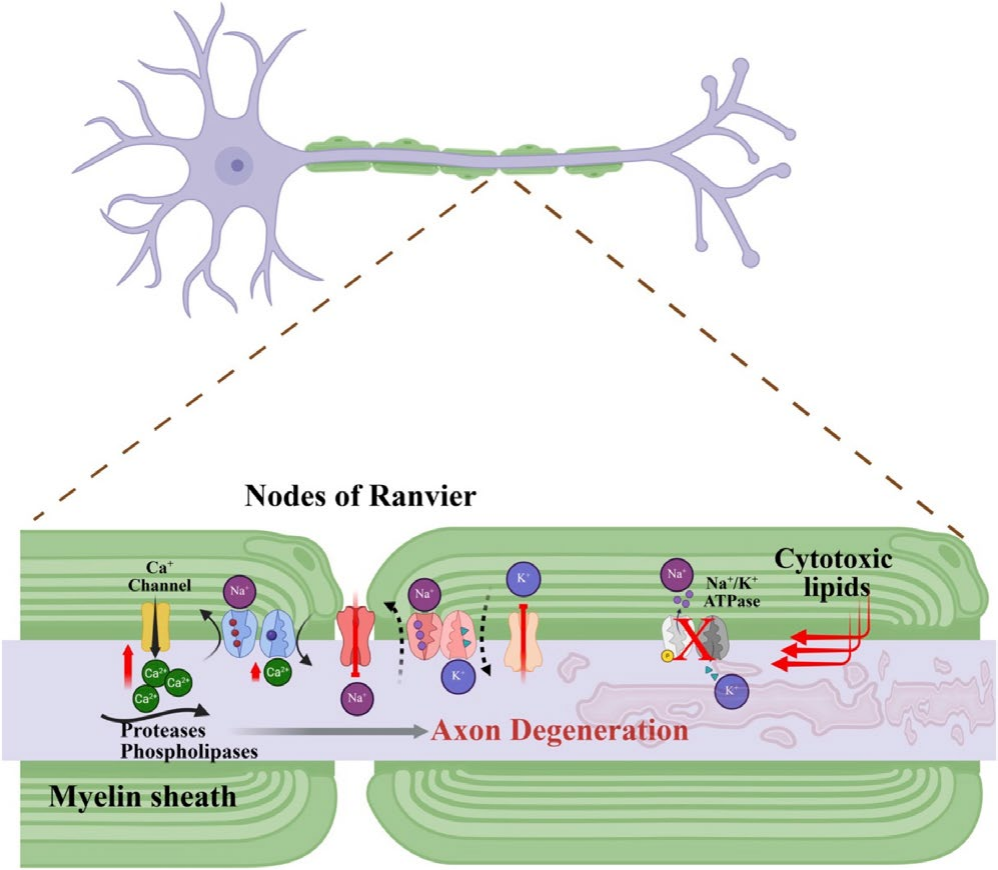
Altered neuronal conduction and signaling can result from lipid‐induced dysregulation of ion channels. Cytotoxic lipids can disrupt Na^+^ and/or K^+^ channels, leading to excitotoxicity. Some lipids impair the Na^+^/K^+^ ATPase, resulting in excess Ca^2+^, which can also occur through impairment of Ca^2+^ channels. High levels of Ca^2+^ activate proteases and phospholipases, ultimately causing axon degeneration.

Lipids exhibit extreme structural and functional diversity, serving as molecules of energy storage, structural components, and dynamic signaling factors. As a result, it is likely that there is not a single mechanism of lipid toxicity in neuropathy, but many, potentially acting concurrently or in a cascade. Additionally, due to the complexity of lipid metabolic pathways, an imbalance in one area of lipid synthesis can often lead to large shifts in cellular lipid metabolism and/or the accumulation of secondary lipid species (Tracey et al. 2018). Due to these complications, in many disorders involving lipid‐induced neurotoxicity/axonopathy, the precise pathological processes are still under investigation. However, reactive lipids, regardless of their source (Schwann cell, oligodendrocytes, immune cells, etc.) have been found to perpetuate several recognized mechanisms of neurotoxicity. These mechanisms are briefly discussed below (Figures 2, 3, 4, 5, 6, 7).

### 6.1 Destabilization of Membrane Function

Lipids constitute the vast majority of both axon and Schwann cell membranes, as a result, many neurotoxic lipids are theorized to disrupt neuron/Schwann cell membrane flexibility, electrical dynamics, and signal transduction (Perez‐Matos et al. 2017). In neurons, the disruption of membrane dynamics can either reduce the ability of axons to propagate action potentials, leading to deficient conduction, or alternatively, excitotoxicity and apoptosis (Taso et al. 2019). Furthermore, altered membrane dynamics in Schwann cells can cause dys/demyelination and disruption of trophic support/intercellular signaling to axons, prompting secondary axonopathy (Schmitt et al. 2015). For example, plasmalogens, which are enriched in myelin, decrease membrane fluidity and promote incorporation of cholesterol compared to other phospholipids (Han and Gross 1991). Notably, mice deficient in the biosynthesis of plasmalogens have peripheral hypomyelination and radial sorting defects, leading to axon degeneration (Da Silva et al. 2014). The biophysical properties of plasmalogens promote the formation and stabilization of lipid rafts, which are major membrane structures enriched in phospholipids, cholesterol, and sphingolipids and serve as instrumental organizing centers for signaling proteins and receptors. They are thought to be particularly sensitive to lipid modifications (Tracey et al. 2018) (Figure 2). Pathological alterations of lipid raft domain function driven by toxic lipid accumulation have been theorized to result either from the direct incorporation of accumulated lipid species or from imbalances in lipid synthesis and transport resulting in a non‐optimal ratio of lipid membrane components (Bonetto and Di Scala 2019; Campomanes et al. 2019). Additionally, lipid peroxidation has been demonstrated to alter the composition of lipid rafts (Mesa‐Herrera et al. 2019). As lipid rafts have been implicated in a wide range of processes including receptor localization/trafficking, neurotransmitter transport, cytoskeletal rearrangement, exocytosis, and metabolic signaling, it is clear that their disruption has widespread effects on neuronal health and survival (Allen et al. 2007).

### 6.2 Oxidative Stress and Lipid Peroxidation

Many neurotoxic lipid species contribute to the initiation or amplification of oxidative stress. Oxidative stress occurs when the accumulation of reactive oxygen species (ROS) in the form of oxidants or free radicals is not mitigated by cellular antioxidants. ROS are highly reactive and cause widespread modifications to other lipids, proteins, and nucleic acids, disrupting organelle function (Figure 3). Mitochondria and the Endoplasmic Reticulum are especially prone to damage from oxidative stress due to their management of high levels of ROS intermediates, leading to further metabolic imbalance (Singh et al. 2019). Additionally, a process called lipid peroxidation occurs when ROS modify the polyunsaturated fatty acids (PUFAs) of cellular membranes, leading to membrane disruption and the generation of highly reactive aldehyde species including 4‐hydroxy‐2‐noneal (HNE) and malondialdehyde (MDA). Once formed, these aldehydes bind covalently to amino acids, a process termed Michael adduction, to form protein adducts, which can cause DNA damage, further organelle collapse, and the initiation of apoptotic signaling (Taso et al. 2019). Several noted cytotoxic lipids are themselves ROS and are either unstable metabolic intermediates or generated from intermediate oxidation and are therefore theorized to contribute to oxidative stress in neurons (Komen et al. 2007; Korade et al. 2010; Nagai 2015; Pfeffer et al. 2016). Additionally, oxidative stress can be elevated through the depletion of cellular antioxidants, such as the depletion of NADPH, if aberrant lipid accumulation causes large‐scale imbalances in energy homeostasis (Cashman and Höke 2015).

Notably, neurons are more susceptible to many toxic lipids than Schwann cells. The reasons for this differential sensitivity are not clear; however, Schwann cells produce enormous quantities of lipids to form myelin (Saher and Simons 2010). Therefore, it is reasonable to expect that mechanisms to cope with toxic lipid metabolites evolved in these cells. Indeed, the basal levels of many antioxidant factors, such as catalase, heme oxygenase, and nuclear Nrf2, are higher in Schwann cells than in sensory neurons (Vincent et al. 2009).

### 6.3 Disruption of Energy Metabolism

While disruption of mitochondrial and ER function prompted by lipid peroxidation is a major factor in metabolic disruption, accumulated lipid species are often tied to large shifts in cellular metabolism. For example, high levels of free fatty acids in diabetic neuropathy or following a high‐fat diet are thought to promote insulin resistance through inducing the formation of diacylglycerols (DAG) and triacylglycerides (TAG) that in turn lead to activation of the PKC (protein kinase C)‐theta and PKC‐delta kinases. These kinases can then phosphorylate and inhibit insulin receptor proximal signals, such as the insulin receptor substrate (IRS), thereby compromising insulin signaling and altering cellular glucose metabolism (Figure 4) (James et al. 2021; Perez‐Matos et al. 2017). Axons are very energy demanding, due to their propagation of action potentials and the need to transport materials to and from the cell body. Therefore, any impediment to glucose metabolism can disrupt a broad range of functions, from mitochondrial transport to ion homeostasis, leading to axon degeneration.

Various lipids can also deplete the levels of nicotinamide adenine dinucleotide (NAD+), a key electron carrier necessary for cellular metabolism and a critical regulator of axon health. For example, in hepatic cells, the fatty acid palmitate was shown to inhibit nicotinamide phosphoribosyltransferase (NAMPT), resulting in depletion of NAD+ (Penke et al. 2017). If a similar pathway is activated in axons, the reduction in NAD+ would stimulate the NADase SARM, which further reduces NAD+, impairing metabolic activity in the axon and leading to degeneration (Waller and Collins 2022).

### 6.4 Biological Activity/Altered Signaling

While lipid molecules were once considered largely structural molecules, it has become clear that many lipids are dynamic regulators of key cellular pathways. They serve as receptor ligands, scaffolding substrates, and secondary messengers in an expansive range of cellular processes, including neurotransmitter release, channel regulation, proliferation, apoptosis, inflammatory signaling, and metabolism (Perez‐Matos et al. 2017; Schmitt et al. 2015; Tracey et al. 2018). Consequently, alterations in either lipid species concentrations or signaling can lead to cytotoxic effects. For example, ceramides are potent regulators of inflammatory signaling factors, and when accumulated, have been shown to propagate apoptotic signaling in neurons through activation of NF‐κB (Gill and Windebank 2000, 1998) and/or c‐Jun N‐terminal kinase (JNK) (Willaime‐Morawek et al. 2003). The activation of JNK can lead to caspase activation (Haimovitz‐Friedman et al. 1997), which not only promotes apoptosis but, when restricted to the axon, induces axonal degeneration (Geden et al. 2019) (Figure 5). Ceramides can also damage mitochondria, leading to ROS production and neurodegeneration (Kennedy et al. 2016). Additionally, lipid species in the same class can have opposing cell functions. For instance, some species of PUFA, including many omega‐3 fatty acids, are known for having anti‐inflammatory signaling, while many omega‐6 fatty acids are pro‐inflammatory (Tracey et al. 2018). Thus, even seemingly small‐scale biogenic imbalances within lipid classes can initiate substantial cellular pathway disruption, potentially leading to neurodegeneration.

### 6.5 Disruption of Axonal Transport

Both motor neurons and DRG sensory neurons feature long axonal projections, containing a disproportionately large cell volume relative to their soma. This results in PNS neurons having a tremendous reliance on cellular transport machinery, both retrograde and anterograde, to support their energetic and biosynthetic requirements. In addition, the extensive transport requirement is itself very metabolically demanding, such that glycolysis can occur directly on transport vesicles to generate local ATP (Zala et al. 2013). If either retrograde or anterograde transport is disrupted, axonal degeneration and cell death can result (Cashman and Höke 2015; Coleman 2005; Stavrou et al. 2020). A variety of lipids, such as ceramides, long‐chain acylcarnitines, saturated fatty acids, and lipid peroxidation products, can disrupt mitochondrial function and energy production. This disruption can hinder the active transport of mitochondria in axons, contributing to degeneration. For example, treatment of DRG neurons with palmitate or stearate reduced the fraction of motile mitochondria and the velocity of their movement, leading to neuronal apoptosis (Rumora et al. 2022). Disruptions in lipid composition on organelles can also impair motor protein activity, leading to deficits in axonal transport. Roney et al. investigated the etiology of neuron degeneration in Niemann–Pick disease type C, a lysosomal storage disorder (Roney et al. 2022). Using a mouse model of the disease, they demonstrated that cholesterol accumulated on lysosomal membranes, sequestering the motor protein kinesin‐1, leading to reduced axonal degradation capacity and, ultimately, degeneration. Moreover, pharmacological reduction of lysosomal cholesterol restored lysosomal transport and reduced neuronal death. Thus, various lipids can promote axon degeneration by impeding the transport of vital axonal components, such as mitochondria and lysosomes.

### 6.6 Ion Channel Dysregulation

All cells are reliant on efficient intracellular and extracellular ion channel function to maintain membrane potential and function. However, neurons are especially sensitive to channel dysregulation as ion channels are fundamental components of neuronal conduction and signaling (Kim 2014). Altered sodium (Na^+^) or potassium (K^+^) channel function can result in either excitotoxic signaling or mechanisms of toxicity related to reduced conduction. Impaired intracellular channel regulation can also initiate or amplify cell stress and organelle dysfunction (Cashman and Höke 2015). Accumulation of intra‐axonal Na^+^ can also occur through lipid disruption of the Na^+^/K^+^ ATPase. Various lipids, including saturated sphingomyelin in the presence of cholesterol, can inhibit this pump leading to excess Na^+^ accumulation (Habeck et al. 2015). The excess Na^+^ gets replaced by Ca^2+^ due to the reversal of the Na^+^/Ca^2+^ exchanger. Axonal buildup of Ca^2+^ results in degeneration due to activation of a variety of catabolic enzymes, such as the protease calpain and phospholipases (Correale et al. 2019).

Neurotoxic lipids have also been implicated in disrupting intracellular Ca^2+^ channels through mis‐localization, altered membrane dynamics, or altered cell signaling (Choi et al. 2015; Wilson et al. 2018). Additionally, some neurotoxic lipids are thought to affect axonal Na^+^ or K^+^ ion channel localization and sensitization through alterations in signaling, membrane dynamics, or obstruction of Schwann cell‐axon communication (Figure 7) (Bonetto and Di Scala 2019; Kleinecke et al. 2017).

## 7 Overview of Diseases Linked to Neurotoxic Lipid Accumulation in the PNS

The brain and spinal cord are uniquely susceptible to disorders of lipid transport and biosynthesis, due to their high energetic and lipid requirements (Corraliza‐Gomez et al. 2019). Therefore, many disorders involving lipid toxicity in the nervous system have severe symptomology in the CNS and are thus more characterized in that context. However, it has long been noted that several primary CNS disorders with toxic lipid accumulation also result in peripheral neuropathy (Chrast et al. 2011; Spassieva and Bieberich 2016). Additionally, there are several neuropathies involving toxic lipid accumulation that are exclusive to the PNS (Atkinson et al. 2017; Schwartzlow and Kazamel 2019). One classic example is Hereditary sensory and autonomic neuropathy type 1 (HSAN1), the most common subtype of hereditary sensory and autonomic neuropathies. It is an autosomal dominant disease caused by missense mutations in the SPTLC1 subunit of serine palmitoyltransferase (SPT), which catalyzes the initial step in the synthesis of sphingolipids (Penno et al. 2010; Rotthier et al. 2010). The disease‐causing variants result in a toxic gain‐of‐function where the enzyme prefers alanine or glycine over the usual serine, resulting in the formation of 1‐deoxysphingolipid products. Because of the missing hydroxyl group from serine, which is needed for their conversion to more complex sphingolipids as well as their degradation, they accumulate in the cell. These 1‐deoxysphingolipids are neurotoxic, particularly to sensory neurons (Penno et al. 2010). However, the mechanisms underlying the neurotoxicity are poorly understood. In addition, the specific cell type where these lipids accumulate is not clear, although given that Schwann cells produce copious amounts of sphingolipids during myelin formation, it is likely that 1‐deoxysphingolipids accumulate in these glia, which could then be released and act on neurons.

**FIGURE 8:**
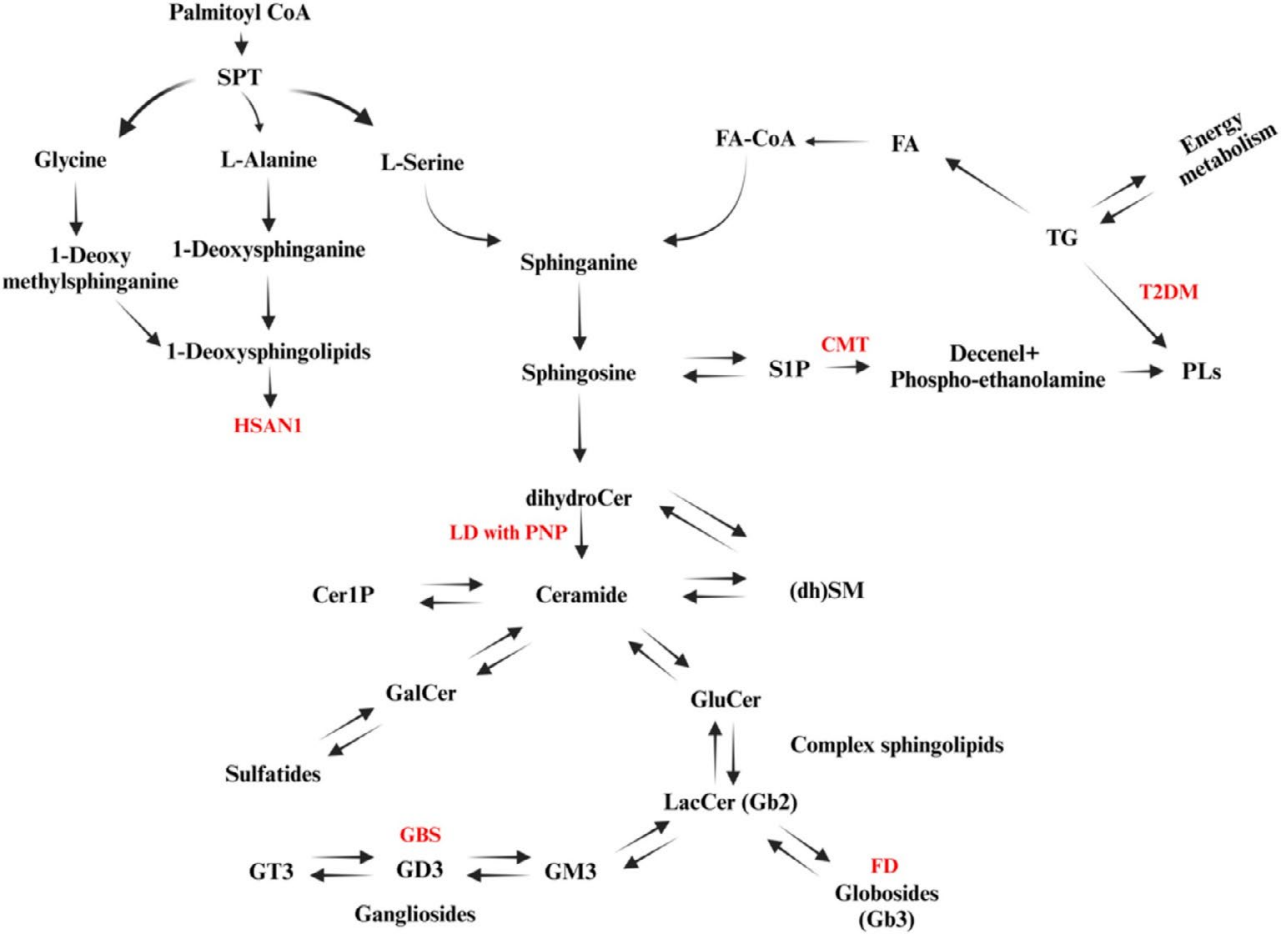
Outline of sphingolipid metabolism and the metabolic steps that were found to be associated with peripheral neuropathies. Cer, ceramide; CMT, Charcot–Marie–Tooth disease; (dh)SM, (dihydro)Sphingomyelin; FA, fatty acids; FD, Fabry disease; GalCer, galactosyl ceramide; GBS, Guillain–Barre syndrome; GD3, disialogangliosides; GluCer, glucosylceramide; GM3, monosialogangliosides; GT3, tetrasialogangliosides; HSAN1, hereditary sensory and autonomic neuropathy; LacCer, lactosylceramide; LD with PNP, leukodystrophy with purine nucleoside phosphorylase deficiency; S1P, sphingosine 1‐phosphate; SPT, serine‐palmitoyl transferase; T2DM, type 2 diabetes mellitus; TG, triglycerides [adapted from (Hornemann 2021)].

Many lipid metabolic disorders are inherited and have known mutations in either lipid biosynthetic enzymes or lipid transporters, resulting in sometimes extreme but also somewhat predictable disruptions in lipid metabolism (Figure 8; Table 1). However, there are many neuropathies where the etiology is either unknown or, as in diabetic neuropathy, may involve irregularities in many intertwined and poorly elucidated lipid‐mediated cell processes. Thus, a better understanding of the complex pathways governing lipid metabolism in both normal development and in disease is needed to inform our inquiries into the causes and potential treatment of neuropathies.

**TABLE 1:** Diseases associated with toxic lipid accumulation.

Disease	OMIM^a^	Pathway affected	Gene	Protein affected	Lipids accumulated
Fabry disease	301,500	Sphingolipid	*GLA*	α‐Galactosidase A	Globotriasylceramide, Globotriasylsphingosine
Metachromatic leukodystrophy, PSAP variant	249,900	Sphingolipid	*PSAP*	Saposin B	Sulphatide, lysosulfatide
Metachromatic leukodystrophy	250,100	Sphingolipid	*ARSA*	Arylsulphatase A	Sulphatide, lysosulfatide
Krabbe disease, atypical	611,722	Sphingolipid	*PSAP*	Saposin A	Galactosylceramide, psychosine
Krabbe disease	245,200	Sphingolipid	*GALC*	β‐Galactosylceramidase	Galactosylceramide, psychosine
Niemann‐Pick (type A, B)	257,200, 607,616	Sphingolipid	*SMPD1*	Sphingomyelinase	Sphingomyelin
HSAN, type 1A	162,400	Sphingolipid	*SPTLC1 SPTLC2*	Serine palmitoyl transferase	Deoxysphinganine, deoxymethylsphinganine
Farber disease	228,000	Sphingolipid	*ASAH1*	Acid ceramidase	Ceramide
AR‐CMT2	N/A	Sphingolipid	*SGPL1*	Sphingosine 1‐phosphate lyase	Sphingosine 1‐phosphate
Adrenoleukodystrophy	300,100	Fatty acid catabolism	*ABCD1*	Adrenoleukodystrophy protein	Very long‐chain fatty acids
Refsum disease	266,500	Fatty acid Catabolism	*PHYH*	Phytanoyl‐CoA hydroxylase	Phytanic acid
Cerebrotendinous xanthomatosis	213,700	Sterol	*CYP27A1*	Sterol 27‐hydroxylase	Cholestanol
Niemann‐Pick (type C, type D)	257,220, 607,625	Sterol	*NPC1, NPC2*	NPC‐1 and NPC‐2	Cholesterol, various sphingolipids
Tangier disease	205,400	Sterol	*ABCA1*	ATP‐binding cassette transporter	Cholesterol esters
Smith‐Lemli‐Opitz	222,100, 125,853	Sterol	*DHCR7*	7‐Dehydrocholesterol reductase	7‐Dehydrocholesterol, 8‐Dehydrocholesterol, various oxysterol metabolites
Diabetes mellitus	270,400	Assorted		Assorted	Assorted

^a^ OMIM, online Mendelian inheritance in man.

## 8 Conclusions

Although it has long been recognized that neurons depend on Schwann cells for their viability, the mechanisms involved remain poorly understood. Nevertheless, a growing body of evidence indicates that Schwann cells play a critical role in maintaining axon integrity and function. It is well known that Schwann cells regulate the formation of axonal structural domains necessary for propagating action potentials; in particular, the formation of the node and surrounding regions, as well as mitochondrial localization and transport within axons. It is also evident that there is extensive communication and metabolic transfer between the Schwann cells and axons, with Schwann cells delivering everything from small metabolites like lactate up to whole ribosomes to the axons. However, the role that these interactions play in regulating axon integrity and function is largely unknown. Schwann cell‐derived metabolites, such as lactate and pyruvate, have been suggested to promote axon function by acting as an energy source for action potential propagation; however, the majority of evidence indicates that axons do not depend on these glial‐derived metabolites for homeostatic activity, but only during repeated firing at a high frequency. In contrast, following a nerve lesion or in pathology, such as diabetes, axons depend heavily on lactate and/or pyruvate from Schwann cells for their integrity and regenerative capacity. Axons are also very sensitive to disruption of Schwann cell metabolism, particularly lipid metabolism. Obstruction of lipid biosynthesis or catabolism in these glia can lead to the release of neurotoxic lipids, which cause axon degeneration. Exactly why such lipids are toxic, particularly to neurons, remains to be determined, although a number of mechanisms have been proposed from inducing oxidative stress to impeding axonal transport. Clearly, a better understanding of the intimate relationship between Schwann cells and their associated axons is essential for understanding overall nerve function and the etiology underlying peripheral neuropathies.

## Author Contributions

Rose Follis wrote the original draft. Vishwanath V. Prabhu and Bruce D. Carter revised the text and created the figures.

## Conflicts of Interest

The authors declare no conflicts of interest.

## Data Availability

Data sharing not applicable to this article as no datasets were generated or analysed during the current study.
